# Metallurgical and Electrochemical Properties of Super Duplex Stainless Steel Clads on Low Carbon Steel Substrate produced with Laser Powder Bed Fusion

**DOI:** 10.1038/s41598-020-67249-2

**Published:** 2020-06-23

**Authors:** Pratik Murkute, Somayeh Pasebani, O. Burkan Isgor

**Affiliations:** 1 Materials Science, School of Mechanical, Industrial and Manufacturing Engineering, Oregon State University, Corvallis, Oregon, USA; 2 Advanced Manufacturing, School of Mechanical, Industrial and Manufacturing Engineering, Oregon State University, Corvallis, Oregon, USA; 3 Advanced Technology and Manufacturing Institute (ATAMI), Corvallis Oregon, USA; 4 School of Civil and Construction Engineering, Oregon State University, Corvallis, Oregon, USA

**Keywords:** Engineering, Materials science, Metals and alloys

## Abstract

This study aims to improve the corrosion resistance of the low carbon steel by cladding it with super duplex stainless steel using laser powder bed fusion process. Critical process parameters such as laser power, laser scan speed, hatch spacing, and powder layer thickness were optimized to achieve the best possible metallurgical bonding between the clad and the substrate. The evaporative losses experienced during the laser melting process resulted in clad layers with lower chromium content (12–25 wt. %) as compared to 26 wt. % of the feedstock powder. A clad thickness of 65.8 µm was achieved after melting ten 50 µm thick powder layers. The higher cooling rates associated with laser powder bed fusion resulted in fine high aspect ratio columnar grain structures with predominantly ferrite grains; however, widmanstätten austenite needles were observed with increasing laser scan speeds. Increasing scan speed had a negative impact on the thickness, corrosion resistance, and the pitting potential of the clads exposed to 3.5 wt.% NaCl aqueous solution. Clads produced at the lowest scan speeds showed comparable corrosion resistance to rolled and annealed super duplex stainless steel.

## Introduction

Duplex stainless steels (DSS) are a special class of ferrous alloys with a balanced ferrite (δ) and austenite (γ) phase microstructure. This dual-phase structure imparts these steels a high strength, toughness, and increased corrosion resistance in environments containing acids, acid chlorides, seawater, and caustic chemicals. Super duplex stainless steels (SDSS)^1^ are a variant of DSS with higher Cr (25–26 wt. %) and Mo (4 wt. %) contents. SDSS typically shows superior pitting and stress corrosion cracking resistance than DSS; therefore, the use of SDSS has been increasing in highly corrosive environments, including oil and gas infrastructure, desalination plants, chemical storage tanks, heat exchangers, and paper/pulp manufacturing facilities^1^. Although low carbon steel (LCS) remains to be the most widely used ferrous alloy, its applications are limited because it is highly vulnerable to corrosion in neutral, acidic, or saline environments^2^. Corrosion-resistant SDSS alloys possess superior corrosion resistance than the LCS for the aforementioned applications, albeit with a significantly higher material cost. One of the viable means to reduce the component cost without compromising on service life is to manufacture a composite with a low-cost tough and ductile substrate cladded with a wear and corrosion resistant surface, as in the case of SDSS clads on an LCS substrate.

These dissimilar metal composites (cladded systems) have been produced in the past using conventional manufacturing techniques such as welding^3–5^, diffusion bonding^6^, powder roll bonding^7^, hot rolling^8^, and reduction bonding^9^. However, conventionally manufactured composites are often unfit for field use due to substandard clad-substrate bonding, often leading to clad delamination when subjected to operational stresses in the field. This substandard bond typically is a consequence of distinct and abrupt metallurgical transition at the clad-substrate interface. Furthermore, these conventional manufacturing techniques often result in a clad layer with imperfections such as porosity, undercuts, crevices, pinholes, and keyholes leading to reduced service life^2,10^.

Recent developments in additive manufacturing (AM) technologies make them a good candidate to produce cladded systems with required properties^11–14^. Traditionally, the AM techniques have employed to produce three-dimensional (3D) components, for which the physical and mechanical properties of the components are dictated by the cohesion between the layers of similar metal. However, the key criteria for a successful and effective cladding operation is a superior clad-substrate bond, which involves adhesion of two or more dissimilar metals. Consequently, the production of 3D components and cladding operations are fundamentally different in regard to the bonding dynamics of materials.

The laser powder bed fusion (LPBF)^15^ is a promising method for cladding operations because of its higher resolution and dimensional accuracy than the other powder bed-based AM technologies such as directed energy deposition (DED), electron beam melting (EBM), and binder jetting^16–19^. Moreover, LENS and DED are not suitable for producing clads with micron-level thickness, primarily owing to its powder feed mechanism. Specifically, these methods result in lower-dimensional accuracy, higher material consumption per unit volume of print, and higher surface roughness of the components produced. Therefore, surface preparation steps are often required after components are printed. Despite its higher dimensional accuracy than LENS or DED, EMB requires high vacuums and pre-heated substrates, resulting in significant energy consumption. On the other hand, LPBF operates at ambient pressures, room temperatures, and has a precise melting mechanism owing to its well-controlled laser and optics systems enabling the user to define the laser spot size for melting. The static powder bed ensures the minimum use of materials for the print without the need for support structures.

In a recent study, LPBF was successfully used to produce 316L-SS clads on LCS substrate^20^. It was shown that the LPBF process that produced low-defect clads with good adherence to substrate required a significantly higher volumetric energy density (VED) (in the range of 333–1333 J/mm^3^) than conventionally required for producing 3D parts (~100 J/mm^3^)^21^. The clads had Cr contents in the range of 13–15%, and hardness higher than that of the annealed and wrought AISI 316L-SS alloy. It was observed that the corrosion resistance and other electrochemical properties of the clads produced at lower scan speeds (e.g., 100 mm/s) were comparable to the wrought alloy^20^, thus establishing the feasibility of LPBF process for cladding operations to produce corrosion and wear-resistant surfaces. Since SDSS shows significantly higher stress corrosion cracking and pitting corrosion resistance than 316L-SS in chloride environments, (e.g., seawater^1^), it is imperative to produce highly corrosion-resistant SDSS surfaces with inexpensive LCS substrates for severe exposure conditions where 316L-SS clads might prematurely fail and not provide adequate service life.

It is fairly established that powder feed AM processes with travelling heating and feedstock source (e.g., DED/LENS) are more versatile than LPBF in producing highly complex parts at higher production rates^11,19,22^. However, the LPBF process has considerable advantages over DED/LENS with respect to energy consumption, powder recyclability, dimensional tolerance and defect count in the built parts^11,23,24^. Among these benefits, those related to corrosion performance are important to highlight. LPBF has considerable advantages over DED/LENS in producing clads with high corrosion performance due to its ability to reduce defects, particularly when the corrosion resistant layer is designed to be rather thin, as in the application presented in this paper. The finer particle size and a wider particle size distribution of the feedstock powder from gas atomization process used for LPBF, result in a highly packed and dense powder bed^25^, resulting in higher density, lower porosity, and low defect counts for LPBF parts as compared to those with DED/LENS for the same unit energy used. Furthermore, DED/LENS processes typically manifest poor dimensional tolerances as compared to LPBF components due to powder blowing on the melt pool in contrast with a localized laser melting on static dense powder bed. LPBF achieves higher dimensional accuracy and surface finish quality than DED/LENS; both of these properties affect corrosion performance. It is widely recognized that surface imperfections are detrimental to corrosion properties, particularly for localized corrosion issues such as pitting and crevice corrosion^26^.

Additional benefits of LPBF involve lower energy consumption^27,28^ and higher powder recyclability^29^ when compared with the DED/LENS processes. Typically, DED/LENS employs high power heating source (electrons/laser) in the kilowatts power range, whereas LPBF uses lasers in the range of few hundred watts^11,19,30^. The DED/LENS processes where a mix of powders are blown on the melt pool, powder recyclability is nearly impossible, whereas the LPBF has near complete recyclability of the unmelted feedstock powder due to very localized melting and lower powder contamination^31,32^.

Other processes such as chemical vapor deposition (CVD), physical vapor deposition (PVD) could be employed for high density coatings/cladding on a nano and micrometer range; however, these processes are limited to a small surface areas with poor process yields^33^. Therefore, LPBF has clear advantage over CVD/PVD for producing large scale micron size-thick clads.

In this study, we report the development of metallurgically-bonded, corrosion-resistant, micron-scale SDSS clads on LCS substrate using LPBF. These corrosion-resistant SDSS clads are intended to provide superior service life than their LCS counterparts and higher pitting and stresses corrosion cracking resistance than traditional stainless steels in chloride-containing aggressive environments. We further characterize the physical, metallurgical, and electrochemical properties of SDSS clads in regards to changing laser scan speeds and energy density. Finally, a comparison of expected service lives of LCS and cladded systems is made based on general corrosion rates showing a significant increase in corrosion performance.

## Results and Discussions

### Clad surface characteristics

**Figure 1:**
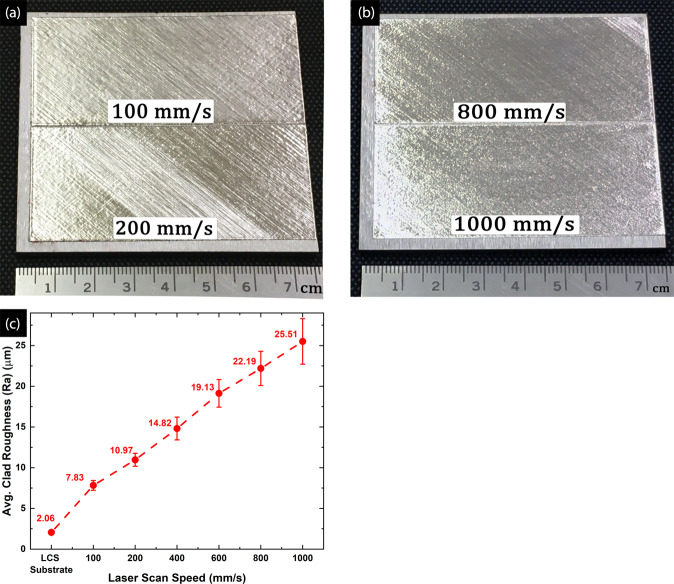
SDSS clads at produced at P = 200 W, h = 30 µm, d = 50 µm and can speeds of (**a**) v_s_ = 100 and 200 mm/s, (**b**) v_s_ = 800 and 1000 mm/s. The clads show increased surface roughness with increasing laser scan speeds, (**c**) Variation of SDSS clad roughness with increasing scan speed.

Amongst all LPBF process parameters, the scan speed (v_s_) has a profound impact on the surface roughness and the dimensional accuracy of the 3D component^34^. For cladding operation in this study, similar observations were made in regard to the clad surface quality. Figure 1 presents the snapshots of the SDSS clads at the lowest (Fig. 1(a)) and the highest (Fig. 1(b)) scan speeds. The clads manifested a relatively smooth surface finish with minimal balling effect at the clad perimeter at all scan speeds (100–1000 mm/s). The minor balling around the perimeter is attributed to the changes in melt orientation causing excessive melt splashing and discontinuity in powder spreading at the edge of the clads. Furthermore, it was observed that the clad surface roughness and balling phenomenon at clad perimeter, increased with increasing scan speed. The balling phenomenon occurs due to a combination of factors including capillary instability, high scan speeds resulting in melt splashing, little liquid content in melt pools resulting from low or inadequate laser volumetric energy density (VED), and Plateau-Rayleigh instability^35^. A detailed discussion on the balling phenomenon and its adverse effects of the component properties are discussed elsewhere^16,21,36–42^.

The average surface roughness values for SDSS clads at different scan speeds and the LCS baseplate are presented in Fig. 1(c). As Illustrated in figure, the average clad surface roughness increased with increasing laser scan speeds. This behavior is consistent with the previous studies dealing with surface characteristics of the AM parts^11,26,36,43^. Furthermore, the standard error associated with the measurements increased with the scan speeds. This increasing roughness trend could be attributed to decreasing melt pool width and decreasing overlap between two adjoining melt tracks, resulting in creating of valley like features between two centers of melt tracks^25,36^. Furthermore, the intense melt pool splashing at higher scan speed results in spatter generation^44^, thereby causing increased surface roughness. Since the LCS substrate was machined to a tight tolerance, it showed the lowest roughness values of 2 µm.

### Clad-heat affected zone profile properties

**Figure 2:**
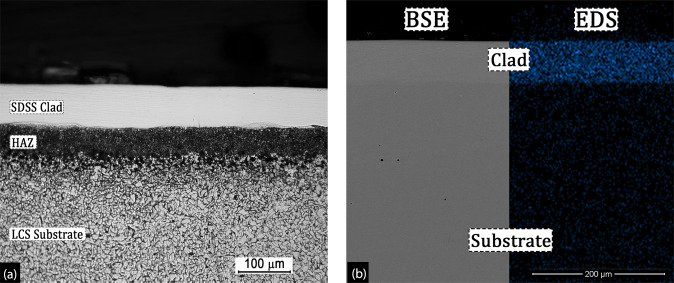
(**a**) the optical micrograph showing the SDSS clad with underlying HAZ in the LCS substrate and (**b**) the backscattered electron mode image showing elemental Cr contrast in the clad layer (left) and corresponding Cr area map of the clad-substrate interface (right)(v_s_ = 100 mm/s and P = 200 W).

As presented in Fig. 2, the SDSS clads at all laser scan speeds were found to be fully dense, without any traces of lack-of-fusion (LOF) porosity or keyhole defects. However, it should be noted that the clads were produced using only 10 layers of printing. Therefore, the defects (LOF/keyhole/balling), once formed would rapidly propagate with successive powder layer spreading and increasing total clad thickness.

**Figure 3:**
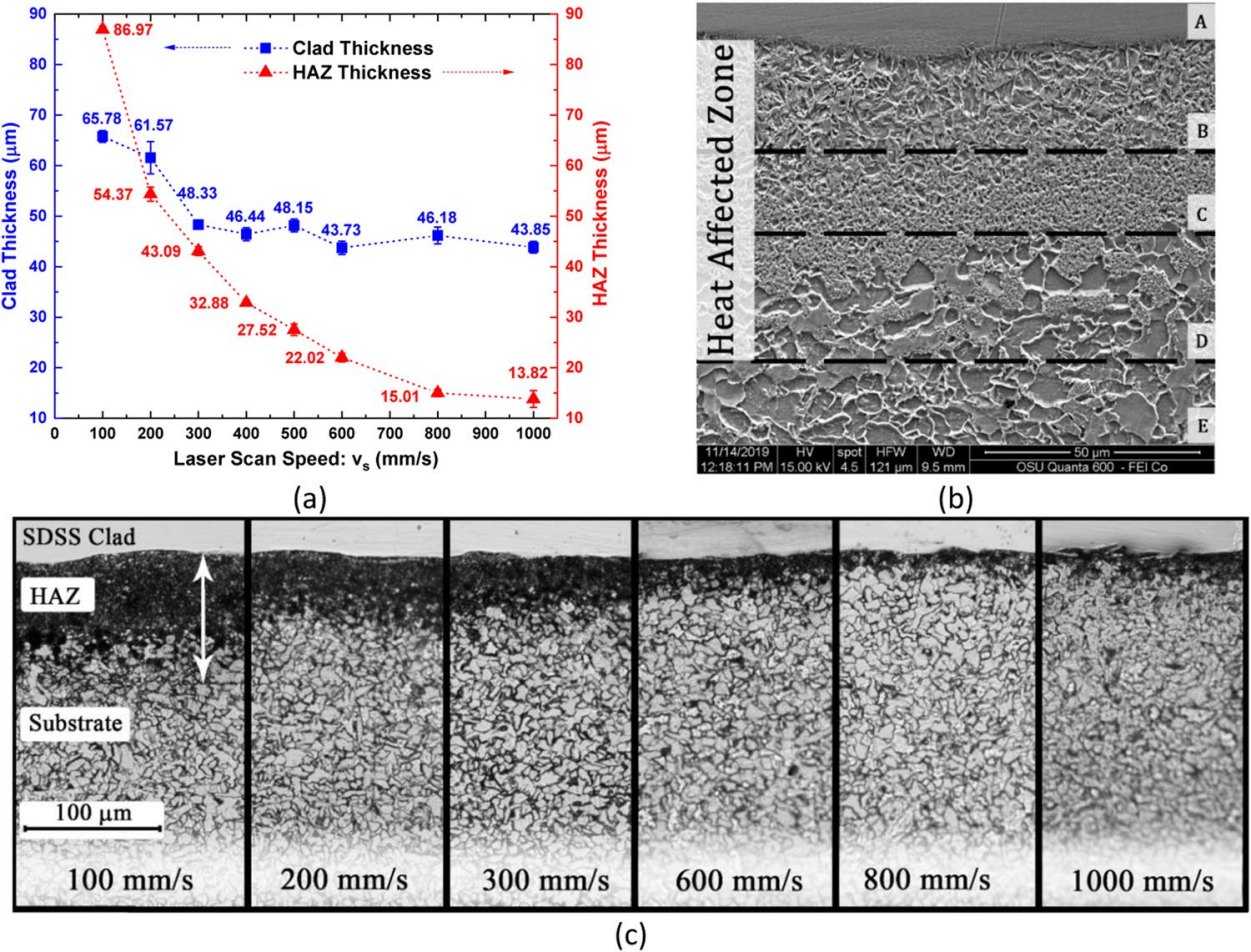
(**a**) Decreasing clad and HAZ thickness with increasing scan speed, (**b**) SEM image of the HAZ underlying SDSS clads showing different microstructures along the HAZ depth and (**c**) representative optical micrographs showing clad-HAZ-substrate regions for SDSS clad system illustrating variation of HAZ thickness with increasing scan speeds.

Figure 2(a) presents the optical micrograph of the clad-substrate interface showing the heat affected zone (HAZ) beneath the SDSS clad formed during to laser melting of feedstock SDSS powder. These optical micrographs were used to measure the clad thickness, and the measurements were validated using the elemental chromium contrast observed in the backscattered electron (BSE) imaging mode of SEM. The Cr contrast was observed due to the difference in chromium content between Cr-rich clads and Cr-poor substrate. Figure 2(b)-left side shows a BSE-SEM micrograph of the representative Cr contrast observed at the clad-substrate interface for clad produced at v_s_ = 100 mm/s. This Cr contrast is presented as EDS elemental area map for the same specimen (Fig. 2(b)-right side). Clad thicknesses were measured at various locations along the width of the cladded sample, and Fig. 3(a) presents the clad thickness plotted as a function of scan speed. It was observed that increasing scan speed had an adverse impact on the clad thicknesses. The maximum average clad thickness of 65.8 μm was obtained at the lowest scan speed of 100 mm/s, whereas the lowest average clad thickness was observed at 1000 mm/s. In general, the average clad thickness decreased with increasing scan speeds. This decreasing clad thickness trend with increasing scan speed can be elucidated on the basis of VED (Eq. 2 described in the methods section), the formation of denudation zones during the laser melting, and the laser - powder bed interaction as discussed in the following sections.

Bidare *et al*.^45^ and Khairallah *et al*.^40^ in their FEM studies on laser-powder bed interaction during localized melting noted that with increasing scan speed, the interaction between laser-plasma plume and the powder bed becomes more pronounced. This intensified interaction causes melt pool instability, which results in increasingly higher amounts of powder (molten and unmelted) being blown away from the melt pool. Furthermore, high melt pool temperatures during laser melting of powder cause the evaporation of metal, resulting in a net upward flux of vapors from the melt pool. This upward flux creates a low-pressure zone at the base of the laser-plasma plume, which due to the pressure differential, results in the flow of the ambient inert gas (present study: N_2_ gas) towards the melt track, and this phenomenon is commonly known as Bernoulli Effect^43,46^. This inward flux of the inert ambient gas is adequate to entrain powder particles from the vicinity of the melt pool, which can then either be ejected with the metal vapor or be incorporated into the melt pool.

As the Bernoulli effect intensifies with increasing scan speed, the width of the denudation zone increases. The denudation zone is the area adjoining the solidified melt track with depleted powder particles^43,45,46^. Consequently, the formation of wider denudation zones due to the increased Bernoulli effect results in the unavailability of feedstock powder for the subsequent melting to form the clad layer at higher scan speeds. This resulted in the melting of powder layer thicknesses lower than what was initially spread (50 μm). The results presented in Fig. 3(a), are in good agreement with the theory of denudation zones, and the Bernoulli effect^43,46^.

In the context of the present study, the heat affected zone (HAZ) (Fig. 3(b)) is the region of the substrate, beneath the clad layer undergoing microstructural changes due to high melt pool temperatures (~5000–7000K^45^) during localized laser melting followed by rapid cooling rates (10^4^–10^6^ K/s) in a N_2_ inert atmosphere. The HAZ at all scan speeds are presented in Fig. 3(c), and the HAZ thicknesses, measured using optical microscopy, are plotted as a function of v_s_ in Fig. 3(a). As illustrated in Fig. 3(a,c), increasing scan speed resulted in decreasing HAZ thickness. Since all other process parameters (P, h, d) were fixed, increasing scan speed from 100 mm/s to 1000 mm/s, resulting in a VED decrease from 1333.33 J/mm^3^ to 133.33 J/mm^3^ respectively, resulting in deeper heat penetration in the substrate at lower scan speed or higher VED. As a result, the microstructural changes in HAZ were observed at greater depths for slow scan speeds, and vice versa.

The HAZ formed during laser welding has been shown to have lower corrosion resistance as compared to the base alloy or the cladding^13,47–50^. This higher corrosion susceptibility of the HAZ, amongst numerous other factors, is primarily due to microstructural effects, decarburization and sensitization. Similarly, for the present work, the HAZ formed below the SDSS clad would be vulnerable to the corrosion attack. When the SDSS-LCS composited are in service, the HAZ would be exposed to the corrosion attack only after the entire thickness of SDSS clad is consumed or in the event of localized defect formation (pinhole/crevice) in the clad resulting in corrosive medium reaching the HAZ.

Consequently, higher clad thickness would effectively delay the exposure of HAZ to the corrosives. Furthermore, higher scan speeds result in higher corrosion rates of the SDSS clads as presented in subsequent section. Therefore, low thickness with high corrosion rates of the clads would prematurely expose the HAZ at high scan speeds, thereby resulting in myriad of corrosion issues. Some major issues such as galvanic coupling, crevice corrosion and HAZ corrosion would significantly deteriorate the service life of component, possibly resulting in catastrophic failure. Therefore, to eliminate the deleterious effect of HAZ, the cladded components would be heat treated before being employed in service. The investigation on the effects of heat treatment on HAZ and clads properties is beyond the scope of the work presented in this manuscript; the heat treatment study is presently underway.

### Microstructural characterization

**Figure 4:**
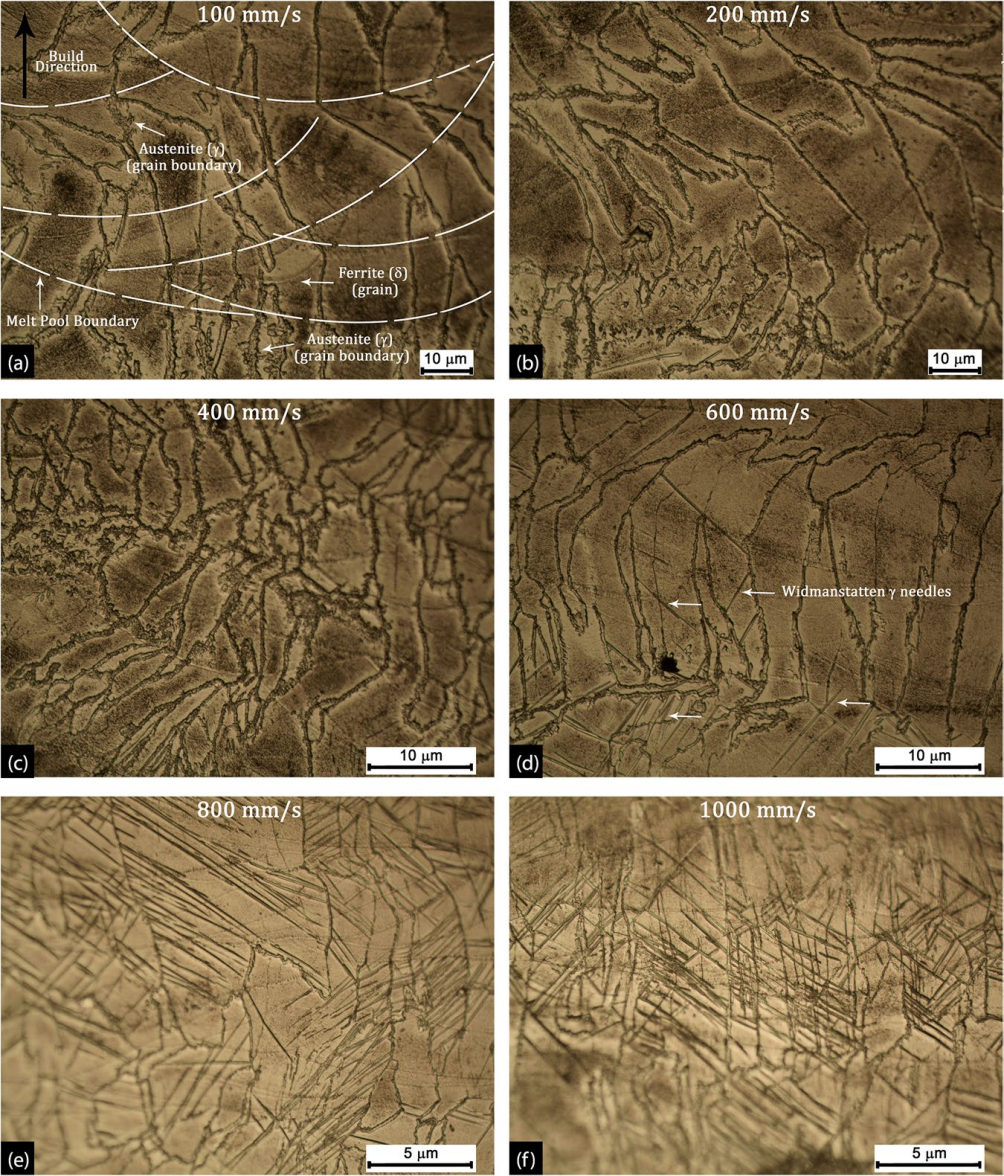
The optical micrographs showing the microstructure of the SDSS clad produced at scan speed of (**a**) 100 mm/s, (**b**)200 mm/s, (**c**) 400 mm/s showing δ- ferrite columnar grains with austenite phase on grain boundary and (**d**) 600 mm/s, (**e**) 800 mm/s and (**f**) 1000 mm/s showing the increasing presence of Widmanstatten austenite (needle/lath microstructure) in δ- ferrite matrix.

The characteristic microstructure of the SDSS clads produced at different scan speeds are presented in Fig. 4. The build direction (black arrow) and the melt pool boundaries (white dashed lines) within the clads are marked in Fig. 4(a). It was observed that clads produced at low scan speed (e.g., @ 100–400 mm/s) resulted in long columnar grains growing normal to the substrate and parallel to the build direction. The rapid unidirectional cooling typically associated with the LPBF process results in a high aspect ratio columnar grain structure with grains transcending across multiple melt pool boundaries. In the present context, unidirectional cooling was a consequence of heat dissipation from the clad layer to the underlying LCS substrate, which served as a heat sink. Furthermore, with increasing v_s_, the grain size decreased due to increasing cooling rates and decreasing VED. As presented in Fig. 4, the 100 mm/s clads showed an average grain area of 108 ± 19 µm^2^, whereas 600 mm/s and 1000 mm/s clads showed an average grain area of 63 ± 12 µm^2^ and 19 ± 4 µm^2^ respectively. These average grain areas in the SDSS clads were significantly smaller than the wrought and annealed base SDSS (578 ± 28 µm^2^). Therefore, LPBF clads has considerably larger, high-energy grain boundary area as compared to conventionally produced base SDSS. SDSS clads are expected to show lower corrosion performance as compared to base SDSS owing to higher grain boundary area, columnar grain morphology and higher residual stresses.

The SDSS clad microstructure showed predominantly ferrite (δ) phase with austenite (γ) precipitation at the grain boundaries at scan speeds (100–400 mm/s), however, at higher v_s_ of 600–1000 mm/s, the presence of Widmanstätten austenite (needle/lath morphology) was observed along with grain boundary γ precipitation. The width of the widmanstätten needle observed in micrographs were in the range of 0.2–0.5 μm. Typically, high cooling rates of LPBF result in high ferrite content (ca. 93–95%) with primary precipitating of austenite at the grain boundary and secondary precipitation in the form of Widmanstätten plates (needle colonies) and inter-granular dispersion. These microstructures are consistently observed in LPBF and welding studies of DSS/SDSS, and the mechanism and the crystallography of the Widmanstätten austenite needles are discussed elsewhere^51,52^.

Furthermore, the presence of austenite decreased with increasing v_s_, and the clads produced at highest scan speeds (800–1000 mm/s) showed predominantly ferritic phase with fine clusters of Widmanstätten γ needles as presented in Fig. 4(d–f). These SDSS clad microstructural observations (Widmanstätten needles and grain boundary precipitates) are in good agreement with previous DSS studies done by Davidson *et al*.^53^, Yang *et al*.^47^ and Hengsbach *et al*.^54^. Similarly, Saeidi *et al*.^55^ via X-Ray diffraction observed a predominantly ferritic microstructure for additively manufactured DSS. Welding studies presented by various researchers have established that under rapid cooling rates, a complete primary solidification from the liquid to δ-ferrite takes place^48,56^. Similarly, for LPBF process which is associated with cooling rates of 10^4^–106 K /s, it can be assumed that the molten SDSS powder would solidify majorly to high-temperature δ-ferrite phase according to general solidification reaction for low carbon ferrous alloys; L → δ + L → δ (for C < 0.11wt.%). Therefore, at higher v_s_, austenite (γ) formation is greatly suppressed owing to such high cooling rates.

Typically, for achieving the desired balance between austenite and ferrite phase post welding and LPBF processes, the SDSS/DSS are usually subjected to solution annealing heat treatments^53,54^. Heating at temperatures above the AC_3_ line in the iron-carbon diagram and followed by water quenching result in δ → γ transformation. A similar transformation was also observed in SDSS clads in this study but very insignificantly, which is reflected by the presence of austenite at the δ-ferrite grain boundary.

At higher VED and clad thicknesses, when the top powder layer is laser melted, the previously fused layers underneath are subjected to reheating due to heat dissipation towards the substrate. These reheating cycles lead to δ → γ transformation at high energy areas such as grain boundaries. The austenite phase on the δ grain boundaries is marked in Fig. 4(a). Apart from the very high-temperature gradients, the evaporative loss of ferrite and austenite stabilizing elements like C, Cr, Mo, Ni, and Fe during laser melting results in the imbalance of δ/γ phase fraction. The elemental loss from the clad region is discussed in further detail in subsequent sections.

The optical micrographs of HAZ areas beneath the clad layer and a higher magnification SEM image of the HAZ are presented in Fig. 3(c) and Fig. 3(b), respectively. Figure 3(b) is marked with five different zones (A-E) based on different microstructures and properties. Zone A and E represent the SDSS clad and the unaffected LCS substrate respectively. Zones B, C, and D represent the HAZ of the substrate with varying microstructures depending on the heating cycles the zones were subjected to during laser melting. Zone B (partially melted zone) often termed at CGHAZ (coarse-grained HAZ) shows a coarse grain size due to prolonged exposure to very high temperatures (between liquidus [1539 °C] and above peritectic temperature [1492 °C]: L + δ ferrite), resulting in grain size increase and upon cooling to room temperature transforms into coarse ferrite (α) and pearlite (α+Fe_3_C) phases. Zone C, known as FGHAZ (fine-grained HAZ) or recrystallized zone, shows a very fine grain structure; this zone typically is exposed to temperatures ranging between AC3 [912 °C] and peritectic temperatures [1492 °C]. This temperature range constitutes only austenite (γ) phase on the iron-carbon diagram for low carbon contents (0.2 wt. %), and this γ phase on rapid cooling to room temperature transformed into fine-grained ferrite (α) and pearlite (α+Fe_3_C) microstructure. Lastly, Zone D, which is known as ICHAZ (intercritical HAZ), shows partial grain refinement. Zone D, during the laser melting process, is exposed to temperatures ranging from AC1 [727 °C] to AC3 [912 °C]. In this intercritical temperature range, on heating, the cementite (Fe_3_C) originally present in LCS substrate transforms to austenite (γ), whereas the ferrite (α) phase remains unchanged. On rapid cooling, this austenite transforms back to pearlite (α+Fe_3_C). Therefore, zone D shows patches of pearlite formed in the ferrite matrix. Furthermore, the ICHAZ could be subdivided into ICHAZ and SCHAZ (subcritical HAZ: exposed to below AC1 temperatures); however, such fine distinctions are difficult in shallow HAZ and processes (Submerged Arc Welding) which generate deep HAZ could reveal such distinct areas.

Typically, the melt pool cooling rate varies linearly with the laser scan speeds which follows the Rosenthal formulation^57–59^

1 $$\frac{dT}{dt}=2\pi k\frac{{v}_{s}}{P}{({T}_{m}-{T}_{0})}^{2}$$

where k is the thermal conductivity (W/mK), v_s_ is the laser scan speed (mm/s), P is the laser power (W), T_0_ is the substrate temperature (K), and T_m_ is the melting temperature (K). Numerous experimental and simulation studies have shown that low laser scan speeds result in deeper and wider melt pools^36,39,60,61^. Increasing melt pool volumes causes slower cooling rates due to low surface-to-volume ratio of the cooling liquid metal, resulting in low radiative cooling. In contrast, at faster scan speeds, shallower and narrower melt pool results in rapid cooling of the liquid melt. Therefore, increasing laser scan speeds lead to increasing melt pool cooling rate resulting in the microstructural and metallurgical features discussed in subsequent sections.

These microstructural changes are found at a higher depth of LCS substrate with increasing laser energy density or decreasing scan speeds due to deeper heat penetration. These HAZ microstructural observations are in good agreement with numerous Laser/welding studies previously carried out, and detailed discussions on HAZ could be found elsewhere^62,63^. The presence of HAZ has been known to degrade the mechanical properties and corrosion resistance of the ferrous alloys; therefore, it is imperative to carry out post cladding heat treatments to eliminate/ mitigate the deleterious effects of HAZ.

### Phase identification

**Figure 5:**
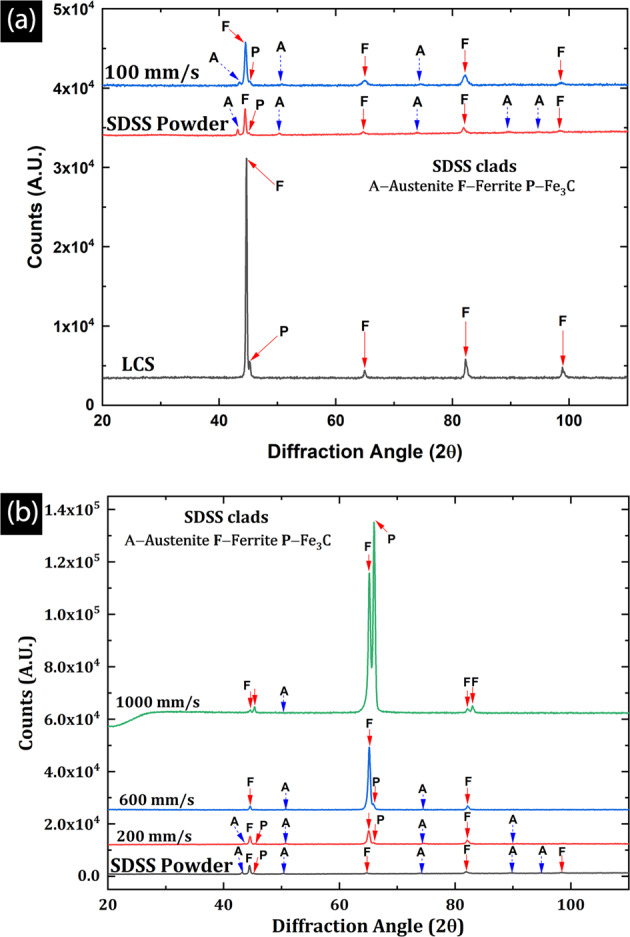
The XRD spectra of (**a**) LCS Substrate, SDSS feedstock powder, and SDSS clads at lowest scan speed (100 mm/s) (**b**) SDSS clads at higher scan speeds (up to 1000 mm/s) showing predominantly ferrite peaks and only lower-order austenite peaks.

The phase analysis of the SDSS feedstock powder, the LCS, and the clads were done using X-ray diffraction (XRD), and the XRD spectra are presented in Fig. 5. As shown in Fig. 5(a), the LCS showed a typical ferrite and cementite (Fe_3_C) peaks, whereas the feedstock powder showed a mix of ferrite and austenite peaks due to its duplex structure. Similarly, the SDSS clad produced at the lowest scan speed (e.g., v_s_ = 100 mm/s) showed peak signatures similar to feedstock powder. All the clad showed a mixture of austenite and ferrite peaks; however, the austenite peak intensity was significantly low as compared to the feedstock powder. This suggests that the austenite phase fraction in the clads is considerably lower than what was initially present in feedstock powder prior to melting. Furthermore, it was observed that the presence of the austenite phase reduced with increasing v_s_, as suggested by the absence of higher-order γ peaks in the XRD spectra at higher scan speeds (e.g., v_s_ = 600, 1000 mm/s). As shown in Fig. 5(b), the clad produced at a scan speed 600 mm/s show predominantly ferrite peaks with only two austenite peaks at 2θ position of 50.80° and 74.32°. However, at the highest scan speed of 1000 mm/s, the clad shows only one austenite peak (50.80°) in the XRD spectra. These results indicate that the formation of austenite is suppressed with increasing cooling rates, resulting in increasing ferrite phase fractions with increasing v_s_. This reaffirms the fact that higher heat inputs (higher VED) or longer dwell times could lead to an improved balance of the ferrite-austenite phase as seen in 100–400 mm/s clads.

The ferrite and austenite phase fractions derived from the Rietveld refinement^64,65^ of the XRD spectra are presented in Table 1. It was observed that increasing laser scan speeds resulted in decreasing austenite and increasing ferrite phase fraction. The SDSS feedstock powder produced via gas atomization showed the highest austenite phase fraction (20.4%); however, with increasing scan speeds, austenite phase fraction decreased with lowest ferrite content of 1.1% in 1000 mm/s SDSS clads. Conversely, the ferrite phase fraction increased from 88.9% at 100 mm/s to 98.7% at 1000 mm/s. These results are in good agreement with the microstructural observations discussed in earlier sections. Typically, for DSS alloys, ferrite phase is more vulnerable to corrosion attack than austenite phase^1,66,67^. Consequently, increasing ferrite content, increasing high energy grain boundary area at faster scan speeds would result in increased corrosion rates as discussed in subsequent sections.

**Table 1:** Ferrite-Austenite phase fraction in SDSS clads at all scan speeds compared with SDSS feedstock powder.

Scan Speed (mm/s)	δ-Ferrite (%)	γ-Austenite (%)
100	88.9	10.7
200	92.2	7.8
400	94.9	5.1
600	95.3	4.5
800	97.7	2.1
1000	98.7	1.1
SDSS Powder	79.6	20.4

**Table 2:** Equivalent circuit modeling parameters after EIS data fitting.

v_s_ (mm/s)	Zphase (min.) (°)	R_ct_ (x10^3^) (ohm.cm^2^)	CCPE (x10^–6^) (ohm^−1^ cm^−2^ s^α^)	α
Base SDSS	−85.34	332.2	30.49	0.95
100	−83.87	297.5	42.94	0.94
200	−80.59	120.7	31.81	0.90
300	−77.05	95.59	31.03	0.86
400	−75.27	26.94	54.96	0.86
500	−78.61	31.23	36.84	0.89
600	−74.32	11.18	76.76	0.88
800	−68.56	6.916	119.6	0.83
1000	−71.34	4.128	104.1	0.87
LCS	−61.71	0.796	318.2	0.86

Increasing ferrite peak intensity at diffraction angles of 65.2° and 65.9° with increasing v_s_ is suggestive of formation of textures in the clads. These textures are related to the high aspect ratio columnar grain growth, preferentially in the direction parallel to the build direction. The detailed investigation of textures is beyond the scope of this article.

### Elemental mapping

**Figure 6:**
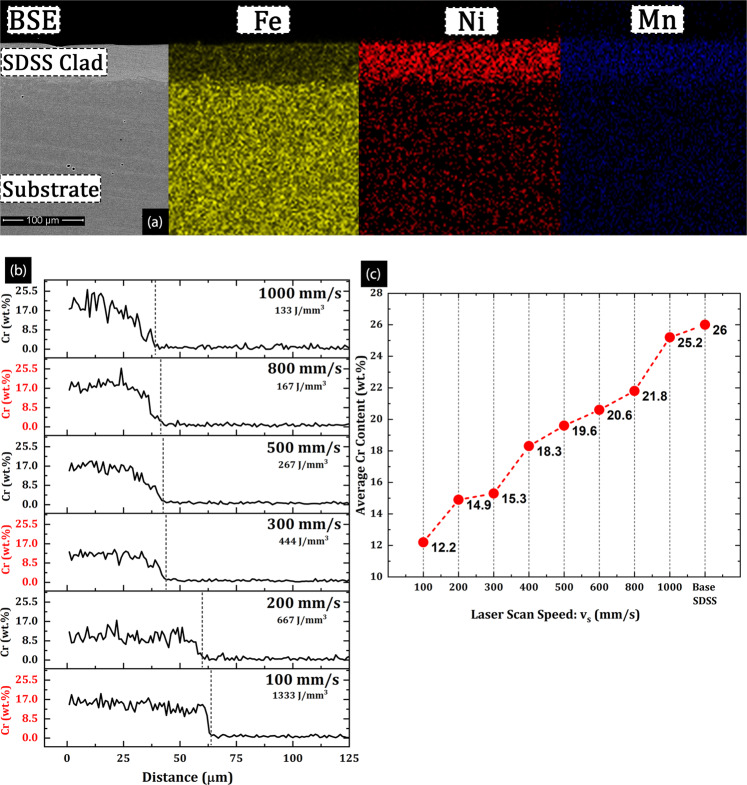
(**a**) The EDS elemental area maps for Fe, Mn, and Ni across the clad-HAZ-substrate, (**b**) elemental line scan for Cr at different scan speeds (black-solid lines) and (**c**) average Cr content in clads plotted as a function of v_s_.

Fig. 6 shows the energy dispersive spectroscopy (EDS) elemental area maps for representative SDSS clads along with corresponding elemental line scans across the clad-substrate interface. As illustrated in the figure, the clads, in general, are rich in Cr (Fig. 2(b)), Ni, and Mn content (Fig. 6(a)), whereas the LCS substrates were Fe rich. However EDS line scans across clad-substrate interface quantitatively showed that at low scan speeds (v_s_ = 100 mm/s), the Cr content in the clad dropped to about 12 wt.% (Fig. 6(b)) which was considerably below the initial Cr content present in the feedstock powder (~25 wt.%) and base SDSS (~26 wt.%) as shown in Table 3. Furthermore, the increasing Cr contents were measured in clads with increasing v_s_, e.g., at 1000 mm/s, clad showed Cr content of ~24.8 wt. %, which was very close to the base SDSS composition, as presented in Fig. 6(c). Bidare *et al*.^45,68^ showed in their FEM simulations that the localized melt pool temperatures reach as high as ~7000 K (6276 °C) depending on the laser power and scan speeds. Since these local melt pool temperatures were higher than the vaporization temperatures of Fe (2860°C), Cr (2670°C), and Ni (2730°C), the elemental loss was experienced due to increased evaporation^69^. Furthermore, lower scan speeds result in higher melt pool temperatures due to considerably longer laser-dwell times; consequently the Cr losses were high at lower v_s_. In the context of cladding operations with any AM techniques, these elemental losses are being reported for the first time such observations have not been previously reported in literature. However, FEM studies by Bidare *et al*.^45^ and Khairallah *et al*.^40^ report the evaporative losses during laser melting in LPBF due to high melt pool temperatures. The vertical dashed lines in Fig. 6(b), marked by a drop in chromium content to a low, consistent level, represent the clad-substrate interface. The clad thickness measurements (Fig. 3(a)) and receding clad-substrate interface, as shown in Fig. 6(b), are in good agreement. Furthermore, it should be noted that all the clads showed a gradual decrease in chromium content going from the clad region to the substrate region; this implied a good mix of clad and substrate materials.

**Table 3:** Nominal chemical compositions of the tested materials (wt. %.).

Material	C	Cr	Ni	Mo	Mn	Si	P/S	Fe
SDSS powder	0.03	25	7.00	4.00	0.9	0.4	<0.04/0.03	
SDSS base	0.03	26	8.00	4.00	1.2	0.8	0.035/0.015	Balance
LCS	0.20	0.7	0.95	0.01	0.8	0.70	<0.04	

An experimental study on LPBF of Ti6Al4V alloys by Hooper^70^ provides a conclusive evidence, that melt pool peak temperatures could reach 4000 K at the VED =40 J/mm^3^. Using numerical modeling and experiments, Du *et al*.^71^ show that LPBF of AlSi10Mg at VED=106 J/mm^3^ could result in melt pool temperatures higher than 3086 K, which is higher than the evaporation temperatures of Fe, Cr and Ni. A separate study by Doubenskaia *et al*.^72^ employed an IR camera to investigate the melt pool temperature profiles during LPBF of 316 L SS (P=30 W, v_s_= 50 mm/s), the results indicated that the melt pool peak temperatures reached 2500 K. A few other works by Ansari *et al*.^73^, and Mirkoohi *et al*.^74^. all point towards the fact that melt pool temperatures would go higher than the vaporization temperature of Fe, Cr and Ni, with the volumetric energy density (VED= 133–1333 J/mm^3^) used in this work. It is to be noted that all the previous studies used significantly lower VED than the present study, therefore it could be asserted with certainty that all the experimental and numerical/FEM simulations are in good agreement with the melt pool temperatures ranging from 5000–7000 K, reaching well above the vaporization temperatures for most elements at VED of 1333 J/mm^3^ used in this study.

The elemental loss from the clads could be assigned to either evaporation during melting or diffusion from clad towards HAZ. The clad thickness measurements as presented in Fig. 3 were performed using optical micrographs, as well as secondary and back-scattered emission modes of SEM. All the three methods resulted in virtually the same average clad thickness, and since the thickness measurement in the backscatter emission mode relies on elemental chromium content, we conclude with a high degree of confidence that the chromium did not diffuse deeply into HAZ.

Furthermore, the high energy AM processes (e.g. DED/LENS) used for cladding and repair application do not show signs of diffusion to the dilution/HAZ zone in the substrate^48,75,76^. Since the DED/LENS are characterized by higher VED and higher melt pool volumes (deeper and wider melt pools) as compared to LPBF, the associated melt pool cooling rates are comparatively lower than those of LPBF. It can be inferred that the amount of elemental diffusion to HAZ/dilution zone in LPBF process with even faster cooling rates would be fairly low.

Typically increasing chromium content in steels relate to increasing corrosion resistance, both general and localized corrosion resistance^2^. Since the chromium content of the clads increase with increasing scans speeds, increasing corrosion resistance is to be expected. However, in case of SDSS clads produced via LPBF, numerous other factors primarily dictate the electrochemical response of the clads. Factors such as grain size, grain morphology, surface roughness, residual and thermal stresses take precedence over the average chromium content while determining the corrosion behavior of the clads.

Apart from the metallurgical factors, residual stresses also play an important role in deciding the corrosion/electrochemical response of the metallic alloy^2^. Components manufactured via LPBF process are typically associated with high residual stresses, and this is caused by small melt pool volumes and rapid cooling rates in inert atmospheres^77–82^. Typically, with increasing laser scan speeds, the melt pool volumes have been shown to decrease, thus resulting higher cooling rates owing to highly pronounced convective and radiative cooling^36,41,61^. Furthermore, it has been fairly established that increasing stresses, either residual or externally applied, result in deterioration of corrosion resistance^83–85^. Therefore, in context of the present study, it has been observed that increasing laser scan speeds have been directly linked with decreasing corrosion resistance of the SDSS clads.

As compared to other AM techniques such as DED/LENS, LPBF has relatively localized melting with lower laser power and smaller laser spot size, resulting in shallower melt pools and higher cooling rates. Therefore, residual stresses associated with LPBF are significantly higher than those generated in DED/LENS processes. However, the quantitative analysis of residual stresses resulting from the LPBF process is beyond the scope of this manuscript.

### Electrochemical characterization

**Figure 7:**
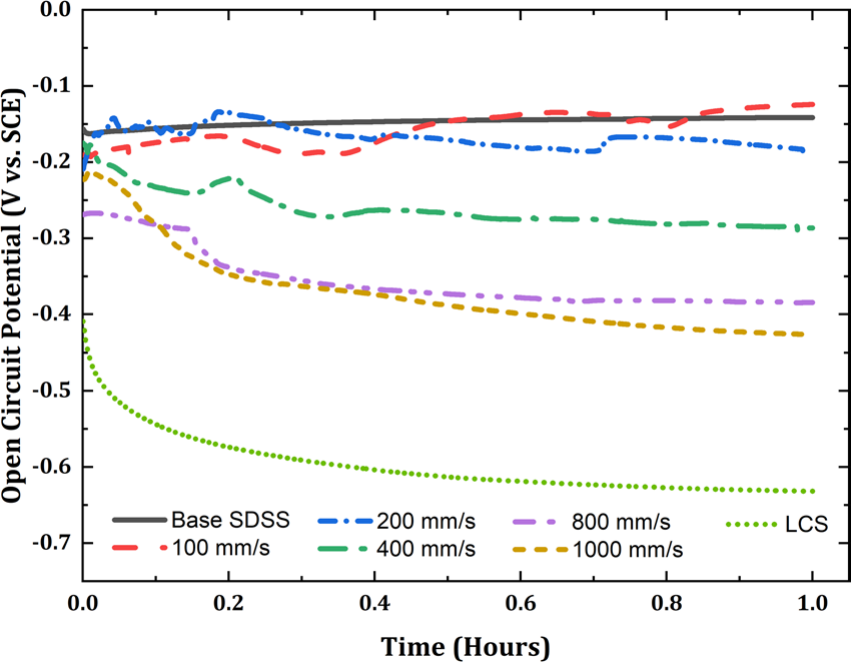
Open circuit potential curves for SDSS clads at all scan speeds.

At the beginning of the corrosion test program, OCP was monitored for one hour. Figure 7 presents the OCP curves for clads, benchmarked with OCP curves of the base SDSS and the LCS substrate. As illustrated in the figure, the base SDSS (− 0.142 V vs. SCE) showed highly positive OCP value as compared to the clads, and the values increased with time because of corrosion-resistant chromium oxide surface film, reducing the corrosion rates to a passive state. In contrast, LCS manifested the most negative OCP value (−0.632 V vs. SCE), which is a typical characteristic of an actively corroding metal. As presented in Fig. 7, all the clad surfaces show a higher OCP value than that of LCS at the end of the exposure period, which suggests that clads were more corrosion resistant than the substrate material. The clad produced at the lowest scan speeds of 100 mm/s (−0.124 V vs. SCE) showed OCP value similar to the base SDSS. However, with increasing v_s_, the OCP values were increasingly more negative for SDSS clads (e.g., OCP_1000 mm/s_= −0.427 V vs. SCE). This decreasing trend of OCP with increasing v_s_ is indicative of decreasing corrosion resistance. The interpretation and implications for this behavior would be discussed in elaborate detail in subsequent sections while discussing electrochemical impedance spectroscopy (EIS), cyclic polarization (CP), and linear polarization resistance (LPR) test results.

**Figure 8:**
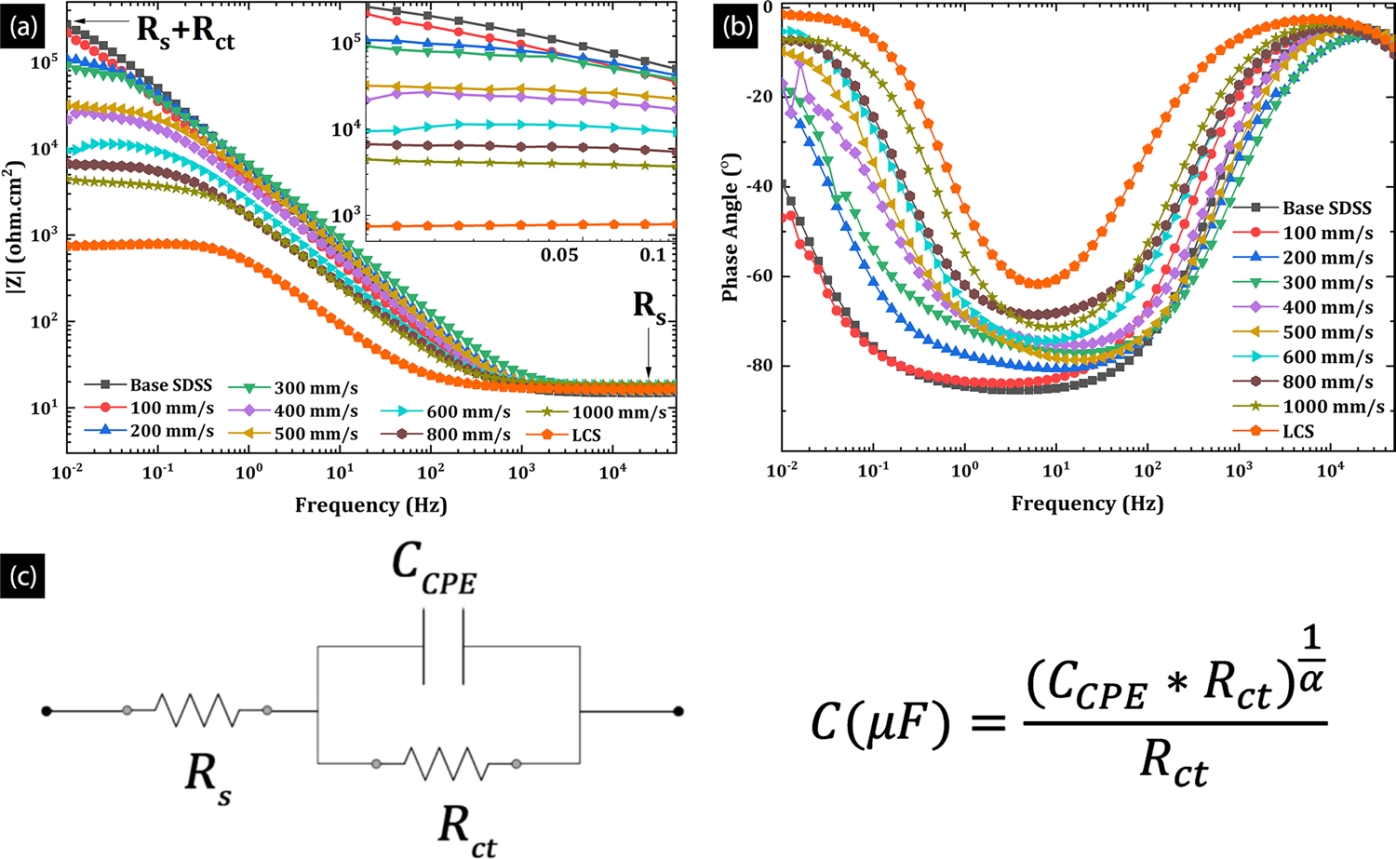
EIS data for the SDSS clads illustrating (**a**) increasing low frequency impedance, (**b**) decreasing phase angle for SDSS clads at various scan speeds (**c**) Equivalent electrical circuit (Randles circuit) used to model EIS data.

Figure 8 presents the electrochemical impedance spectroscopy (EIS) data as impedance bode plot (a), and phase angle bode plot (b) for SDSS clads. The high-frequency impedance values (e.g., at 10^4^ Hz) presented in Fig. 8(a), represent the solution or electrolyte resistance (R_s_); 17.5 ohm.cm^2^ for the 3.5 wt. % NaCl aqueous solution, whereas the low-frequency impedance values (e.g., at 0.01 Hz) indicate the sum of the R_s_ and the charge transfer resistance (R_ct_). The R_ct_ represents the electrical resistance to the charge movement offered by the electrical double layer formed on the alloy surface. Typically the corrosion resistant alloys with protective films exhibit high charge transfer resistance. The R_s_ + R_ct_ value for the LCS substrate (7.5 ×10^2^ ohm.cm^2^) was three orders of magnitude lower than base SDSS (2.7 ×10^5^ ohm.cm^2^), confirming the superior corrosion resistance of base alloys over LCS. The low-frequency impedance values of the clads produced at the lowest scan speed (v_s_ = 100 mm/s) were comparable to base SDSS. However, it was observed that the impedance values were negatively impacted by increasing v_s_; the clads produced at higher v_s_ (e.g., 1000 mm/s: 4.47 ×10^3^ ohm.cm^2^) showed lower impedance value by two orders of magnitude than the clads produced at 100 mm/s scan rate (2.2 ×10^5^ ohm.cm^2^). This behavior is likely because of the higher VED, resulting in higher melt pool volumes and temperatures. Large melt pool volumes result in slower cooling rates, thus promoting the stress relief, alleviated surface roughness, and grain coarsening in the clad layer^36^. These manifold gains because of low scan speed elucidate the higher impedance values observed at 0.01 Hz, as shown in Fig. 8(a). Moreover, for increased cooling rates at higher v_s_, the fine columnar grains with Widmanstätten austenite needles result is large grain boundary area and higher defect density, and with surface roughness due to balling results in lower R_ct_ values for high speed clads.

Figure 8(b) presents the phase angle bode plots for the base SDSS, clads, and the LCS substrate. As illustrated, the base SDSS showed highly negative peak phase angles, in the range of −80° to −85° for a wide range of frequencies(10^–1–^10^2^ Hz), suggesting a near-ideal capacitive behavior of the electrical double layer formed on the alloy surface (ideal capacitor indicated by a phase angle of −90°). On the other hand, LCS showed the lowest phase angles in the range of −60° to −63° in a narrow range of frequencies (~ 5–10 Hz), which is a characteristic of a weak electrical double layer and an unprotective oxide film formation. The clad produced at the lowest scan speed of 100 mm/s showed phase angles similar to the SDSS base. However, increasing v_s_ has a negative impact on the phase angle, i.e., the phase angle increased with increasing v_s_, and eventually, the clad produced at highest scan speeds of 1000 mm/s (SDSS clads) showed comparable phase angle values to LCS (Table 2). The chromium oxide layer forming on the base alloy manifests a very strong capacitive electrical double layer forming on the surface, resulting in a highly negative phase angle value. However, the chromium oxide film forming on the clads are not as dense and protective as base alloys; partly due to reduced Cr content (due to evaporation), high defect density inherent to rapid cooling rates and increased surface roughness, due to increased melt pool splashing and inherent melt pool morphology associated with LPBF process. Therefore, the electrical double layer forming on the clad surfaces is not as strong and, as a result, deviates from the ideal capacitive behavior, leading to increased phase angle values. Since the defect density and surface roughness increases with increasing vs, the non-ideality increases resulting in phase angle increase with increasing vs.

The equivalent circuit modeling of the EIS data was done by fitting the data to a standard equivalent electrical circuit. The electrical circuit used for the data fitting in this study was the simplified Randles circuit (SRC) presented in Fig. 8(c), and the details on the SRC specifics are presented elsewhere^86–88^. This SRC is widely used for modeling of corroding or passivating metal surfaces to quantify assumed electrical circuit elements and their respective physical representations^86–88^. The constant phase element represents the non-ideal capacitive behavior of the electrical double layer forming at the metal/electrolyte interface, which is quantified by a constant phase element - capacitance term (C_CPE_) and an exponent (α)^89^. The relationship between the ideal capacitor and the constant phase element is presented adjoining the randles circuit in Fig. 8(c). Note that α=1 represents an ideal capacitor, and α=0 represents an ideal resistor. The α value close to 1 indicates strong passivity of metal, and therefore, the lower α value denotes deviation of ideal capacitive behavior and is typically indicative of a less passive or actively corroding system.

Table 2 presents the results of the equivalent circuit modeling for the electrochemical data of the SDSS clads. The CPE exponent α value ranged from 0.9 to 1 for base SDSS and clad produced at scan speeds of 100 mm/s and 200 mm/s. The α for v_s_ = 300 mm/s and higher clads were close to LCS (~0.86). The highest R_ct_ value was observed for the base alloys and subsequently clads that were produced at v_s_ =100 mm/s. The R_ct_ decreased considerably for higher v_s_, which is suggestive that they are less protective as compared to the clads produced at v_s_ =100 mm/s. This implies that the ease of charge or ion transfer across the electrical double layer increased with increasing v_s_, resulting in decreased surface impedance and increasing phase angle.

**Figure 9:**
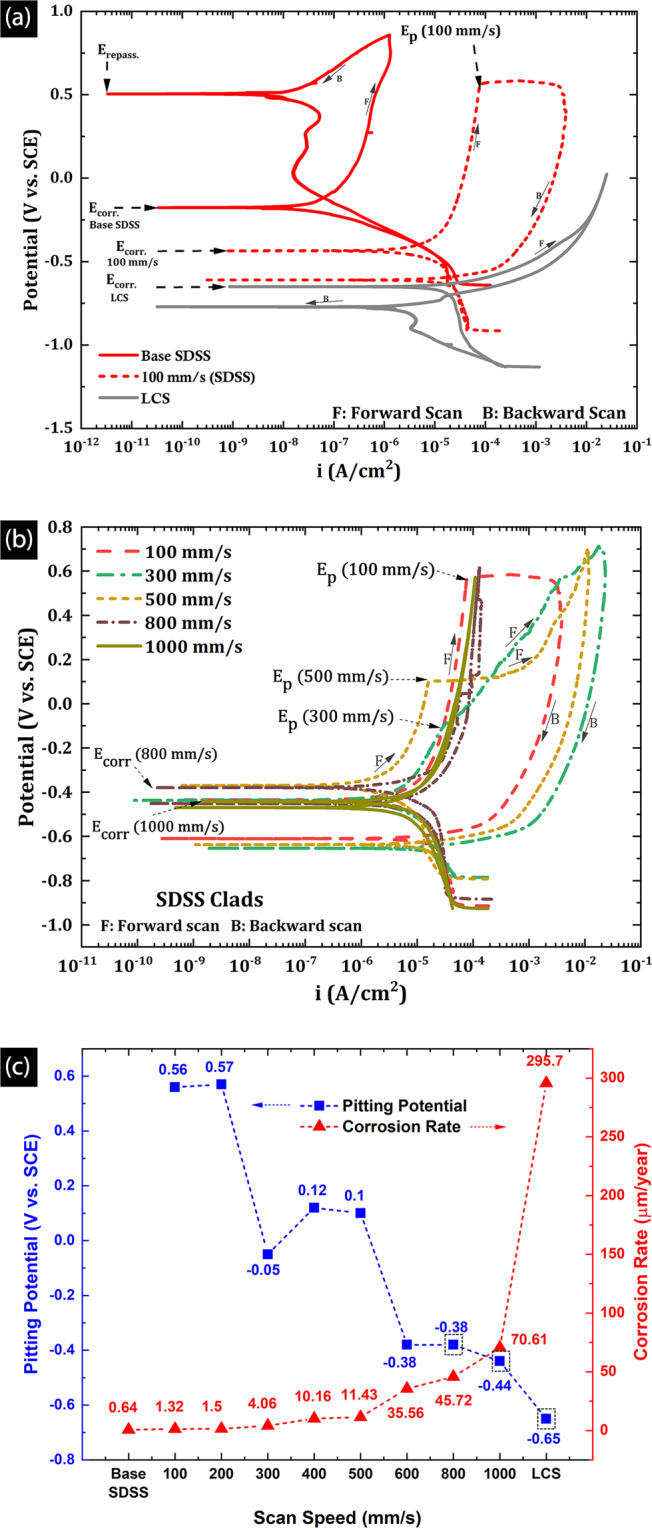
Cyclic polarization curves for (**a**) low speed clads benchmarked with base SDDD and the LCS, (**b**) with increasing scan speeds (**c**) change in pitting potential and general corrosion rate (LPR method) with increasing scan speed.

Figure 9(a,b) illustrates the cyclic polarization (CP) curves for the base SDSS, clads, and the LCS substrate. As shown in Fig. 9(a), the base SDSS does not pit in 3.5 wt. % NaCl solution, owing to its high Cr, Ni, and Mo contents (PREN > 40), also LCS did not show a distinct pitting potential. Therefore all the pitting potentials of the clads would be compared with the corrosion potential of the LCS (Ecorr = −650 mV vs. SCE). Although the clads produced at the lowest scan speeds of 100 mm/s showed a lower pitting potentials than the base SDSS alloys (Fig. 9(a)), their Ep values were higher than the clads produced at higher scan speed and the Ecorr of LCS substrate. Figure. 9(b), illustrates the CP curved for the SDSS clads produced at different v_s_. The SDSS clads produced at higher scan speeds (e.g., v_s_ = 800 and 1000 mm/s) showed signs of metastable pitting but did not show a distinct pitting potential, this is likely due to higher potential sweep rates (0.166 mV/s) for CP tests, due to which the pitting potential was not detected. Therefore, instead of pitting potentials (Ep), their corrosion potentials (Ecorr) are reported in the CP plots. As shown in Fig. 9(c), the increasing v_s_ had a negative impact on the pitting resistance of the clads as represented by decreasing pitting potentials. Higher pitting potentials indicated a stronger resistance to pitting. The boxed markers are the Ecorr values for the aforementioned cases. At this point, it should be noted that Ecorr values followed a similar decreasing trend with increasing v_s_, as pitting potentials.

The difference between E_corr._ and E_p_ values between the LCS and chromium containing specimens (i.e., clads and base SDSS) is due to the chromium oxide layer on the clad/base alloy surface. However, the iron oxides that form on the LCS in neutral chloride-containing aqueous mediums are thermodynamically unstable; thus, unprotective^90^, rendering the carbon steels, high corrosion rates. Additionally, the difference in pitting potentials between clads and base alloys could be attributed to numerous factors such as surface roughness, chemical composition, and different microstructures resulting from different processing techniques. At the outset, base SDSS had a higher chromium content than clads, as shown in Table 3 and Fig. 6(b,c). However, the chromium content of all the clads (~12–25%) was higher than typically required for good corrosion resistance^2^. Consequently, microstructural and surface roughness aspects need to be considered for further understanding the impact of v_s_ on pitting resistance.

The base SDSS was produced via the conventional route of casting, which involved slow atmospheric cool, resulting in a coarse-grained microstructure as presented in Fig. 9(b). However, all clads show columnar grains because of high cooling rates associated with radiative cooling of small melt pool volumes in an inert nitrogen atmosphere (10^4^–10^6^ K/s). On comparing grain sizes, base alloys show coarsest grain size, followed by high aspect ratio relatively coarser columnar grain size in clads and finally the LCS with the finest grains. Since the high-defect and high-energy grain boundaries serve as pit nucleation sites, finer columnar grains resulting in higher grain boundary area in the clads could partially explain the lower Ep of the clads than base SDSS. Finer columnar grains in addition to increasing Widmanstätten austenite needle colonies result in larger grain boundary area in the clads with increasing v_s_, thereby negatively impacting the pitting corrosion resistance.

The presence of nitrogen in the duplex stainless steels has a positive impact on the pitting corrosion resistance^91^ and also has been shown to be a strong austenite stabilizer. Since the clads were produced in N_2_ atmosphere, there is a possibility of nitrogen dissolution in the clads during melting process. Past literature shows an increasing dissolved nitrogen content in the AM component with increasing VED^92,93^. Therefore, increasing VED and slower cooling rates would facilitate nitrogen dissolution in the clads. Since nitrogen is an austenite stabilizing element^94^, the dissolution of nitrogen would results in increasing austenite phase in SDSS. Therefore, it could be predicted that lower scan speed clads would have higher nitrogen content, thus, negatively impacting the corrosion resistance of the clads. It is well established that increasing nitrogen content in steels aids the corrosion resistance^91^. If the nitrogen was dissolved in DSS, it would dissolve up to the solubility limit of N_2_ in austenite (2.4 wt. %). No evidence of nitrides was found in the clads, therefore it is expected that all the dissolved N_2_ will be in the austenite matrix and would not pose any serious threat to the corrosion performance of the SDSS clads by forming any nitrides, which would adversely reduce the pitting resistance of the DSS clads.

Furthermore, the base SDSS with highly polished surfaces had considerably less pit nucleation sites apart from the susceptible grain boundaries. Contrarily, LPBF printed parts have surface defects, volumetric defect density, and roughness due to non-uniform melt pool morphology caused due to melt pool splashing, increased marangoni flow and increased plateau/Rayleigh instability at higher scan speeds. As previously discussed, increasing vs aggravates the balling, thereby increasing surface roughness. The high surface roughness of the clads increased pit nucleation sites and thereby lowering pitting potential below the base SDSS. As shown in Fig. 9(c), decreasing pitting potential resulted from increasing surface roughness and decreasing grain size with v_s_.

The corrosion rates for the base SDSS, the clads, and the LCS were measured by the linear polarization resistance tests. Figure 9(c) presents the increasing trend of corrosion rates (μm/year) with increasing v_s_. The clads produced at the slowest scan speeds (v_s_ = 100 mm/s) exhibited the lowest corrosion rates comparable to the base SDSS. Moreover, the rates were two orders of magnitude lower than that for LCS. Furthermore, the increasing corrosion rates with v_s_ confirms the observations made in OCP, EIS, and CP tests. In terms of the service life of the clads, considering the maximum clad thickness and the lowest corrosion rate for the 100 mm/s clad, the estimated service life of the SDSS clad would be ~ 49.5 years, which is a significant improvement over the service life of carbon steel. During this period (i.e., 49.5 years), as compared to 65.8 µm (v_s_=100 mm/s) of SDSS clad thickness, the LCS would lose 14.65 mm thickness, assuming the measured corrosion rate of 296 μm/year. Furthermore, after the clad is completely consumed, the carbon steel would be exposed, and the service life would depend on the thickness and corrosion rate of the carbon steel used as a structural component. In effect the SDSS clads adds to and increase the service life of carbon steel component significantly.

## Conclusions

In summary, the LPBF technique was successfully employed to produce corrosion resistant SDSS clads on LCS substrate, and following conclusions were drawn.

1. Increasing laser scan speed negatively impacted the clad layer thickness. A maximum clad thickness of 65.8 µm was achieved after printing ten 50 µm-thick powder layers, and the smallest clad thicknesses were observed at the highest scan speed for SDSS clads.
2. The HAZ thickness decreased with increasing scan speeds. Metallurgical characterization revealed the formation of three distinct zones in HAZ; CGHAZ, FGHAZ, and ICHAZ, each with its characteristic microstructures. CGHAZ showed coarse grains of ferrite(α) and pearlite at shallower HAZ depths, FGHAZ showed fine perlite structure at higher HAZ depths, and ICHAZ showed partial grain refinement showing patchy pearlite in ferrite matrix.
3. The clads show a lower chromium content than their corresponding feedstock powders due to evaporative losses experienced due to high VED. However, the clad chromium contents were found to be increasing with increasing scan speeds. Furthermore, all the clads had adequate chromium content (>10.5 wt. %) that was higher than typically required for good corrosion resistance.
4. The lowest scan speed clads (100 mm/s) showed a mix of ferrite and austenite phase; however, with increasing laser scan speeds, the ferrite phase fraction increased, with 1000 mm/s clads showed dominantly ferritic crystal structure with strong textures forming parallel to build direction.
5. Low speed clads showed large δ-ferrite columnar grains with high aspect ratio growing parallel to the build direction with austenite phase precipitating at the ferrite grain boundary. Additionally, high speed clads (v_s_> 600 mm/s) showed austenite precipitation as Widmanstätten needle colonies. The columnar grain size and the needle cluster size decreased with increasing scan speeds.
6. Increasing scan speeds resulted in decreasing corrosion resistance of the clads. The low scan speed clads (v_s_=100 mm/s) showed a comparable corrosion resistance to the rolled and annealed SDSS and significantly higher corrosion resistance as compared to LCS. All clads had highly positive pitting potentials. Although the base SDSS showed no signs of pitting, the pitting on clads was explained on the basis of increased surface roughness leading to an increase in pit nucleation sites on the clad surface.

### Future work

The annealing and heat treatment procedures of the SDSS would change the phase proportion in the clad and relieve the stresses arising from rapid cooling rates, resulting in enhanced corrosion resistance with better metallurgical and microstructural properties of the clads. Therefore, additional works are underway to attain better surface finish and service life of the LPBF printed clads. These studies involve increasing clad thickness and post-printing heat treatment.

## Methods

### Materials

#### Feedstock powder-clad

**Figure 10:**
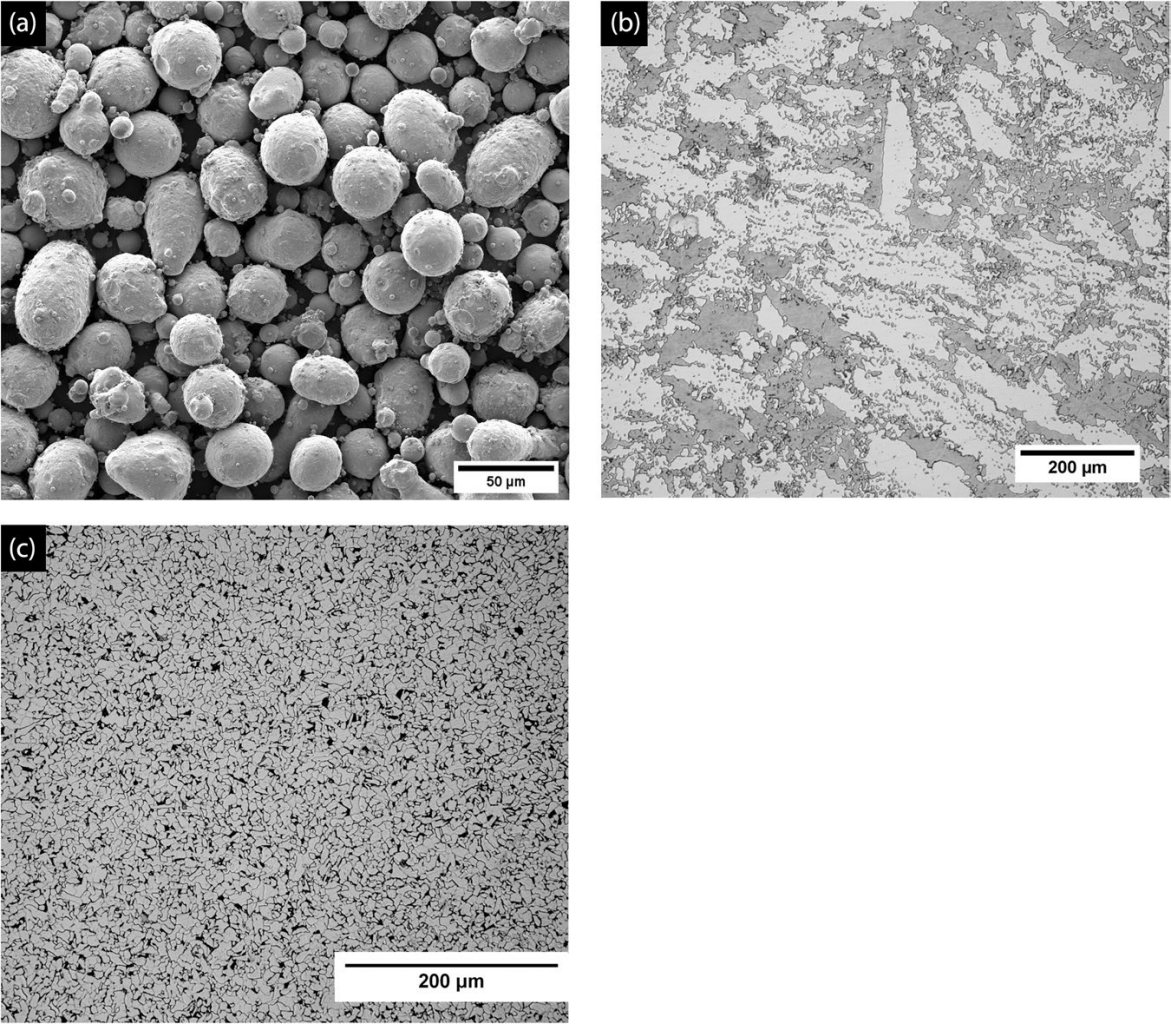
BSE-SEM micrographs of (**a**) gas atomized SDSS feedstock powder (**b**) base SDSS microstructure showing large austenite islands (bright phase) in ferrite matrix (dark phase) and (**c**) LCS microstructure showing fine grains of α- ferrite (bright phase) and pearlite (dark phase).

The present study uses a gas atomized super duplex stainless steel (SDSS) powder (SAF 2507) with a particle size distribution (PSD) of 10–45 μm (D50=25 μm) as a feedstock powder for clads. The backscattered secondary electron (BSE) micrograph showing a typical spherical morphology for the gas-atomized SDSS is presented in Fig. 10(a).

#### Substrate and base alloys

A 1018 low carbon steel (LCS) plate with dimensions 76.2 ×76.2×3.2 mm^3^ was used as a substrate material for the clads. To benchmark the metallurgical and corrosion properties of the clads with traditional alloy, a rolled and annealed UNS32750 alloy (base SDSS) was used in this study. The base SDSS microstructure showed large austenite islands (bright phase) in the ferritic matrix (dark phase) (Fig. 10(b)). Figure 10(c) presents the microstructure of the LCS substrate comprising of α-ferrite (bright phase) and pearlite (α+Fe_3_C) - (dark phase) fine-grained structure. The nominal chemical compositions of the feedstock SDSS powder, base SDSS, and the LCS substrate are presented in Table 3.

### Sample preparation

#### Optical microscopy

For the metallography of the base SDSS and the LCS substrate, all the samples were ground up to 2000 grit size SiC abrasive paper, and cloth polishing up to 0.05 μm sized alumina particle water suspension, and lastly, the specimen was degreased by ultrasonic cleaning in an isopropanol bath for 5 minutes. The metallographic surface preparation procedures were in accordance with the ASTM standard guidelines^95^. To reveal the microstructure, the base SDSS was electrochemically etched with a 50% KOH solution at 5 V for 10 seconds, and the LCS was chemically etched with 2 vol.% nital solution. To investigate the clad microstructure, the clad-substrate composite was epoxy cold mounted to expose the transverse face showing clad-substrate interface. Followed by the cold mounting process, the specimens were ground using silicon carbide paper to 2000 grit size, and cloth polishing up to 0.05 μm sized alumina particle water suspension, and lastly, the specimen was degreased by ultrasonic cleaning in an isopropanol bath for 5 minutes. To reveal the clad microstructures, higher potentials as compared to base SDSS had to be applied (10 V) for 15 seconds. To observe the heat affected zones (HAZ) formed beneath the clad layer, the mounted composite was chemically etched in with 2 vol. % nital solution.

#### Scanning electron microscopy

To determine the morphology and the PSD of the feedstock powder using SEM imaging, a thin layer of feedstock powder was evenly spread on adhesive carbon tape, and any excess powder was blown off the analysis. For the elemental analysis and thickness measurements of the clads, the polished cold mounted composite was sputtered with conductive Au-Pd coating before the SEM analysis. The sputtering provided for better electron grounding and improved imaging.

#### Electrochemical testing

The base SDSS and the LCS surfaces were ground to 2000 grit size of SiC emery paper, followed by buffing and microfiber cloth polishing using a water-based suspension of 0.05 µm sized alumina particles to achieve a mirror finish. But, for the clad surfaces, all electrochemical tests were performed on as printed clad specimens, and therefore all the electrochemical tests accounted for the intrinsic clad surface roughness and defects resulting from the LPBF process. Before electrochemical tests, all the clad surfaces were degreased using ultrasonic cleaning in an isopropanol bath for 5 minutes.

### LPBF Cladding process

All the cladding operations were performed using the OR-CREATOR LPBF 3D-printer. The print chamber was equipped with a 250 W continuous wave Yb- fiber laser (λ=1070±10 nm) and a precision F-θ lens, for accurate laser positioning. At the start of the cladding operation, the print chamber was evacuated, and nitrogen purged, and the clads were produced in an environment with less than 0.1 vol. % O_2_ concentration. The LPBF process typically involves three major stages: (1)The rotating coater blade collected the powder from the feed reservoir and spread it evenly on the print platform (LCS substrate), and the surplus powder was pushed into the excess powder reservoir. (2) After the powder was evenly spread, the laser locally melted the powder in accordance with the 3D CAD file uploaded to the printer interface software. (3) After the melting of one layer, the print platform sunk down by a predetermined layer thickness, and the whole process repeated over, thus producing a 3D part.

For cladding purposes, the printing process was stopped after 10 layers, whereas traditional 3D prints would go from a few hundred to a few thousands of layers, therefore increasing the number of printed layers would only increase the clad thickness. All clads were printed at the laser power of P = 200 W, with laser scan speeds (v_s_) ranging from 100 to 1000 mm/s. The hatch spacing (h) was set at 30 µm, and hatch orientation at 45° w.r.t the base plate orientation. Hatch spacing is defined as the center to center distance between two melt tracks laid subsequently next to each other. The powder layer thickness (d) was set at 50 µm, and since only 10 layers were printed, the total thickness powder layers was 500 µm. These parameters resulted in volumetric energy density (VED) in the range of 133.3 to 1333.3 J/mm3 following:

2 $$VED=\frac{P}{{v}_{s}\ast h\ast d}$$

These print parameters have been fine-tuned for best-clad surface finish and superior clad-substrate bonding and are derived from extensive LPBF process optimization experiments, and the details of these trails could be found elsewhere^20^.

### Microscopy

#### Optical microscopy

A Leica DM2500 optical microscope was used for the clad and the HAZ microstructural analysis post chemical and electrochemical etching. Furthermore, the optical microscopy was used to measure the clad layer and HAZ thickness.

Scanning electron microscopy

The scanning electron microscopy (SEM) was used for the elemental analysis across the clad-substrate interface and for clad thickness measurement. A Thermo Scientific FEI quanta 600 FEG-SEM was used for the backscattered electron (BSE) imaging and energy dispersive X-ray spectroscopy (EDS) line and area scans. Elemental line scans probed into the chemical composition across the clad-substrate interface, whereas the area scans gave the elemental distribution over a larger area of interest, which included clad-HAZ-substrate regions. The backscattered imaging used a voltage of 15–30 kV and spot size of 4.0

### Roughness measurement

SDSS clad surface roughness measurements were done using a stylus type Mitutoyo JIS 2001 surface profilometer. All roughness measurements were performed on a granite surface plate with flatness tolerance of 0.000057”. The stylus moved across the clad surface at 0.5 mm/s.

### X-ray Diffraction

The X-ray diffraction scans of the SDSS powder, LCS substrate, and the as printed clad surfaces were done using Bruker-AXS D8 Discover, X-Ray diffractometer. A copper target operating at 40 kV and 40 mA was used for X-ray source (K_α_ radiations: λ=1.54 Å). All the X-ray scans were performed with the diffraction angles range (2θ) set in the range of 20° − 110° with a step size of 0.05° and a scan speed of 4°/min.

### Electrochemical tests

The corrosion properties of the base SDSS, LCS substrate, and clads were investigated using various open circuit and potentiodynamic AC and DC electrochemical methods. All the electrochemical tests were performed in a three-electrode electrochemical cell with a test electrolyte of a near-neutral 3.56 wt. % NaCl (0.6 M) aqueous solution. The graphite plate and saturated calomel electrode (SCE) served as counter and reference electrodes respectively, and the working electrode was the cladded specimen. A surface area of 2.85 cm^2^ was exposed to the test electrolyte during the tests. The tests were performed using a Gamry 3000 potentiostat/galvanostat/ZRA, and the electrochemical test program comprised of sequentially performed open circuit potential (OCP) monitoring, electrochemical impedance spectroscopy (EIS), linear polarization resistance (LPR) tests, and cyclic polarization (CP) tests. The test sequence started with the OCP monitoring for 1 hour. The OCP monitoring was followed by an EIS scan in the frequency range of 50000–0.01 Hz with an AC perturbation potential of 5 mV r.m.s. After EIS, the LPR test was run with potential scan from −15 mV vs. OCP to +15 mV vs. OCP, and the potential scan rate was set to 0.166 mV/s as recommended by ASTM G61 standard^96^. At the end of the test program, a CP scan was performed with an initial and final potential of −0.5 V vs. OCP and the maximum potential of +1.0 V vs. OCP with a potential scan rate of 0.166 mV/s^96^. A peak current of 20 mA/cm^2^ was set for all the tests. All the electrochemical data presented in this paper were area normalized, and the equivalent circuit modeling of the EIS data was done using Gamry Echem Analyst software (V 6.04).
